# Plasma Periostin Levels Are Increased in Chinese Subjects with Obesity and Type 2 Diabetes and Are Positively Correlated with Glucose and Lipid Parameters

**DOI:** 10.1155/2016/6423637

**Published:** 2016-05-22

**Authors:** Yuanyuan Luo, Hua Qu, Hang Wang, Huili Wei, Jing Wu, Yang Duan, Dan Liu, Huacong Deng

**Affiliations:** Department of Endocrinology, The First Affiliated Hospital of Chongqing Medical University, Chongqing 400016, China

## Abstract

The purpose of this study is to examine the relations among plasma periostin, glucose and lipid metabolism, insulin resistance and inflammation in Chinese patients with obesity (OB), and type 2 diabetes mellitus (T2DM). Plasma periostin levels in the T2DM group were significantly higher than the NGT group (*P* < 0.01). Patients with both OB and T2DM had the highest periostin levels. Correlation analysis showed that plasma periostin levels were positively correlated with weight, waist circumference (WC), body mass index (BMI), waist-hip ratio (WHR), fasting plasma glucose (FPG), 2 h postchallenge plasma glucose (2 h PG), glycated hemoglobin (HbA1c), triglyceride (TG), total cholesterol (TC), fasting insulin (FINS), homeostasis model assessment of insulin resistance (HOMA-IR), TNF-*α*, and IL-6 (*P* < 0.05 or 0.001) and negatively correlated with high-density lipoprotein cholesterol (HDL-C) (*P* < 0.001). Multiple linear regression analysis showed that TG, TNF-*α*, and HOMA-IR were independent related factors in influencing the levels of plasma periostin (*P* < 0.001). These results suggested that Chinese patients with obesity and T2DM had significantly higher plasma periostin levels. Plasma periostin levels were strongly associated with plasma TG, chronic inflammation, and insulin resistance.

## 1. Introduction

Dyslipidemia, characterized by elevated triglyceride (TG), low levels of high-density lipoprotein cholesterol (HDL-C), and the abundance of low-density lipoprotein cholesterol (LDL-C) particles, is quite common in patients with type 2 diabetes (T2DM) [1]. While higher levels of TG in both the fasting state and the nonfasting state are considered the dominant lipid abnormality in insulin resistance and play a pivotal role in determining the characteristic lipid profiles of diabetic dyslipidemia [2], the efforts to understand the pathogenesis of T2DM are increasingly focusing on the disordered lipid metabolism. Recent studies show that changes in lipid profiles may not merely be the consequences of impaired glucose metabolism but also be one of the risk factors accounting for it [3].

It is known that both lipid and glucose are involved in energy metabolism and can be regulated by the liver, which plays a pivotal role in maintaining energy homeostasis during fed-fasting transitions. Studies have shown that the liver can produce several secreted proteins such as fibroblast growth factor 21 (FGF21) [4], *α*2-HS-glycoprotein (Fetuin-A) [5], and pancreatic-derived factor (PANDER) [6] to help regulate hepatic and systemic glucose and lipid metabolism. In addition, the overproduction of hepatic and intestinal lipoprotein can trigger hypertriglyceridemia and increase the levels of free fatty acids which may induce insulin resistance through downregulating the intracellular glucose-6-phosphate levels. At the same time, the tight-knit partners of TG including long chain acyl-coenzyme A (LCCoAs), diacylglycerol (DAG), and ceramides could also interfere with insulin action. Thus, the liver may act as a middle coordinator that bridges the hypertriglyceridemia and insulin resistance [7].

Periostin, originally cloned from a mouse osteoblast cell line [8], is a highly conserved extracellular matrix protein and is implicated in the pathophysiology of tumor development, arthritis, atherosclerosis, and inflammatory diseases [9]. Recent studies also report a link between periostin and metabolic diseases via the JNK-mediated suppression of fatty acid oxidation in the liver [10]. The abnormal expression of periostin in the liver is reported to be closely associated with glucose and lipid metabolic disorders and obesity in an obese mouse model. The fatty acid oxidation resulting from the dyslipidemia can upregulate the content of TG in the liver, while the hyperactivation of the JNK pathway contributes to the development of hepatic steatosis, obesity, and insulin resistance [11]. Thus, the associations between plasma periostin levels and metabolic disorders merit further investigation.

The aim of the present study is to explore the relations among plasma periostin levels, glucose and lipid metabolic parameters, and inflammatory biomarkers in Chinese obese and newly diagnosed T2DM patients.

## 2. Methods

### 2.1. Study Subjects

A total of 161 volunteers were selected from outpatients at the First Affiliated Hospital of Chongqing Medical University (Chongqing, China) between June 2014 and July 2015. The 75 g oral glucose tolerance test (OGTT) was performed, and a standard questionnaire was administered. Participants were classified into groups as having either normal glucose tolerance (NGT) or newly diagnosed T2DM according to the diagnostic criteria of the World Health Organization in 1999. Obesity was defined as having a BMI (body mass index) ≥25 kg/m^2^ according to the WHO-Western Pacific Region diagnostic criteria (2000). None of the patients had received any treatment including diet, exercise, and medication before the present study. The eligible subjects were ultimately categorized into four subgroups for further analysis: the NGT-normal weight subgroup (NW), the NGT-obese subgroup (OB), the T2DM-NW subgroup, and the T2DM-OB subgroup.

Subjects were excluded from the study based on the following criteria:

1. acute and chronic complications of diabetes;
2. smoking and drinking history;
3. hepatic disease (nonalcoholic fatty liver disease, virus hepatitis, and autoimmune hepatitis) or renal and cardiovascular disease;
4. history of tumor and fracture or sustained hypertension;
5. acute and chronic infectious diseases or allergic diseases, and those taking medications that could affect their inflammatory status within 3 months;
6. systemic corticosteroid treatment;
7. women who were currently pregnant, breastfeeding, or taking contraceptive pills.

The present study was approved by the Ethical Committee of the First Affiliated Hospital of Chongqing Medical University. Signed informed consent forms were obtained from all study participants.

### 2.2. Clinical and Biochemical Evaluations

Anthropometric parameters such as body weight, height, waist circumferences (WC), hip circumferences (HC), and blood pressure (BP) were measured in all subjects according to standard protocols. BMI was defined as the weight per height squared (kg/m^2^). The waist-hip ratio (WHR) was calculated as the ratio of the waist circumferences and hip circumferences. A fasting blood sample was collected after an overnight fast (>8 hours), and another blood sample was collected after 2 hours as part of the OGTT. Plasma samples were obtained from the blood samples by centrifugation at 4°C and kept at −80°C until use, and all the samples were analyzed within 3 months. Glucose was assayed using the glucose oxidase method. Glycated hemoglobin (HbA1c) was determined using the method of high-performance liquid chromatography (VARIANTTM II and D-10*™* Systems, BIO-RAD, USA). Fasting insulin (FINS) was measured using an autoanalyzer (ARCHITECT i2000SR System, Abbott Laboratories, IL, USA). Lipid profiles and liver and kidney functions were detected using a biochemical autoanalyzer (ARCHITECT c16000 System, Abbott Laboratories, IL, USA). The homeostasis model assessment insulin resistance (HOMA-IR) was calculated as follows: HOMA-IR = Fasting insulin level (*μ*U/mL) × Fasting plasma glucose level (mmol/L)/22.5.

### 2.3. Assessment of Plasma Periostin, Tumor Necrosis Factor *α* (TNF-*α*), and Interleukin- (IL-) 6 Levels

Plasma periostin, TNF-*α*, and IL-6 levels were determined by the enzyme-linked immunosorbent assays (ELISA) according to the manufacturer's instructions (Human ELISA kit, Wuhan USCN Science Co., Ltd., China). The intra-assay coefficient of variation was 10%, and the interassay coefficient of variation was 12%. No significant cross-reactivity and interference were observed.

### 2.4. Statistical Analysis

All statistical analyses were performed using SPSS version 19.0 (IBM, Armonk, NY). Data were presented as the mean ± SD. Variables with a skewed distribution including HOMA-IR and BMI were log-transformed before statistical analysis. Student's *t*-test and analysis of variance (ANOVA) were used for group comparisons, while the interrelatedness of variables was analyzed using the Pearson correlation coefficient. Partial correlation analysis was performed after controlling for parameters including age, sex, and BMI. Multiple regression analysis was also performed to determine the risk factors associated with the plasma periostin levels. *P* < 0.05 (two tailed) was defined as statistically significant.

## 3. Results

### 3.1. The Clinical Characteristics

The anthropometric and metabolic parameters in different subgroups are shown in Table 1. Between the NGT group and the T2DM group, there were no significant differences in age, systolic blood pressure (SBP), height, weight, and BMI. Compared with the NGT group, the T2DM group had significantly higher levels of diastolic blood pressure (DBP), WC, WHR, total cholesterol (TC), LDL-C, TG, fasting plasma glucose (FPG), 2 h postchallenge plasma glucose (2 h PG), HbA1c, FINS, TNF-*α*, IL-6, and HOMA-IR, whereas the levels of HDL-C were significantly lower in the T2DM group than in the NGT group (*P* < 0.05 or 0.01). Weight, WC, HC, BMI, and WHR and the levels of FINS, HOMA-IR, TG, IL-6, and TNF-*α* were significantly higher in the NGT-OB subgroup than in the NGT-NW subgroup, whereas the levels of HDL-C were significantly higher in the NGT-NW subgroup than in the NGT-OB subgroup (*P* < 0.05 or 0.01). Compared with the NGT-OB subgroup, the levels of DBP, FPG, 2 h PG, HbA1c, FINS, TC, IL-6, and HOMA-IR were significantly higher in the T2DM-OB subgroup (*P* < 0.05 or 0.01). Compared with the T2DM-NW subgroup, the weight, WC, HC, WHR, and BMI, as well as the levels of DBP, HbA1c, FINS, HOMA-IR, LDL-C, TG, and TNF-*α*, were significantly higher in the T2DM-OB subgroup, whereas the HDL-C levels were significantly higher in the T2DM-NW subgroup than in the T2DM-OB subgroup (*P* < 0.05 or 0.01).

### 3.2. Plasma Periostin Levels in Different Groups

**Figure 1:**
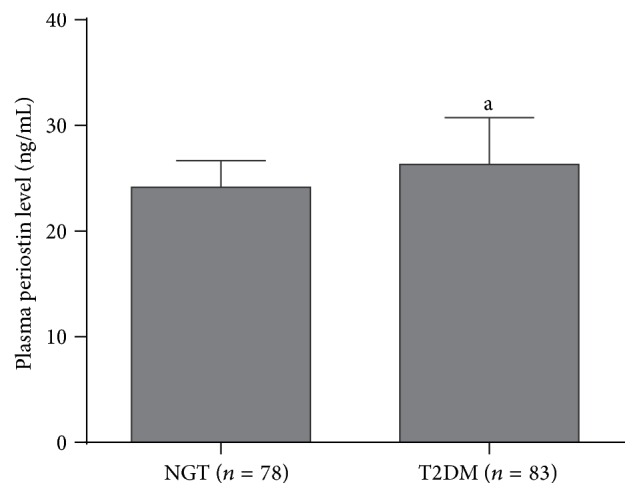
Plasma periostin levels in the NGT group and the T2DM group. The data are presented as the means ± SD. The *t*-test was performed to compare the groups. ^a^ *P* < 0.001 versus NGT.

**Figure 2:**
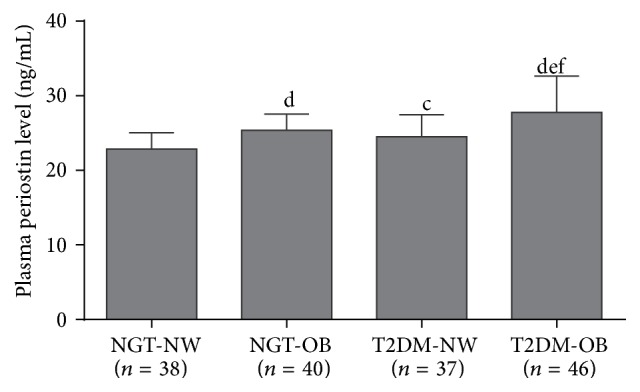
Plasma periostin levels in the four subgroups. The data are presented as the means ± SD. ANOVA was performed for multiple comparisons. ^c^ *P* < 0.05 and ^d^ *P* < 0.01 compared with NGT-NW; ^e^ *P* < 0.05 compared with NGT-OB; ^f^ *P* < 0.01 compared with T2DM-NW.

There were no significant differences in plasma periostin levels between men and women (24.78 ± 3.90 ng/mL versus 25.71 ± 3.61 ng/mL, *P* = 0.119). Compared with the NGT group, the T2DM group had significantly higher plasma periostin levels (26.33 ± 4.41 ng/mL versus 24.16 ± 2.51 ng/mL, *P* < 0.001; Figure 1). Patients with both OB and T2DM had the highest plasma periostin levels (Figure 2).

### 3.3. Relationships between Plasma Periostin Levels and Parameters of Metabolism and Inflammation

As shown in Table 2, correlation analysis showed positive associations between plasma periostin levels and parameters including WC, WHR, BMI, TC, TG, FPG, 2 h PG, FINS, HOMA-IR, IL-6, and TNF-*α* (*P* < 0.05 or 0.001). The plasma periostin levels were negatively correlated with HDL-C levels (*P* < 0.001). After controlling for age, sex, and BMI, the positive correlations between periostin levels and biomarkers including FPG, 2 h PG, TG, TC, FINS, HOMA-IR, TNF-*α*, and IL-6 and the negative correlation of plasma periostin levels with HDL-C still remained significant (*P* < 0.05 or 0.001). Stepwise multiple regression analysis showed that TG, TNF-*α*, and HOMA-IR were independently related to plasma periostin levels (all *P* < 0.001).

## 4. Discussion

Periostin (encoded by* Postn*) is a 90 kDa secreted protein composed of a signal peptide and 4 fasciclin-1 domains [12]. Its name is derived from the fact that it was first detected in periosteal osteocytes and osteoblasts [13]. Later studies found its widespread expression in tissues that are rich in collagen, such as heart valves, tendons, ligaments, and the cornea [14]. Recent studies in both animal and human models show that periostin is involved in the pathophysiology of metabolic diseases. In this study, we found that Chinese patients with obesity and T2DM have significantly higher plasma periostin levels than healthy subjects and that periostin is strongly associated with TG metabolism, chronic inflammation, and insulin resistance.

In an obese mouse model, Lu et al. [10] found that the overexpression of periostin in the liver induced hepatic steatosis and hypertriglyceridemia via JNK pathway-involved downregulation of PPAR*α*, which predominantly regulates the fatty acid oxidation in mitochondria and peroxisomes [15, 16], whereas genetic knockout of periostin or administration of a periostin-neutralizing antibody significantly improved these induced conditions. In our study, we found that patients with obesity had significantly higher levels of periostin. Moreover, periostin levels were positively correlated with TG and TC and negatively correlated with HDL-C, and TG was an independent risk factor influencing plasma periostin levels, after controlling for age, sex, and BMI. These results in humans are in accord with the mouse study mentioned above, and a close relation may exist between periostin and lipid disorders, especially TG metabolism.

The main components of metabolic syndrome are insulin resistance, central obesity, hyperglycemia, and dyslipidemia [17]. Because TG have emerged as the preferred storage nutrient for buffering against fluctuations in energy demand and availability, dysregulated TG accumulation is closely related to obesity and metabolic syndrome [18, 19]. Thus, the underlying mechanism of periostin in the regulation of the metabolic pathway needs further exploration to clarify its clinical points against dyslipidemia, diabetes, and obesity. Thakali et al. [20] found that maternal obesity before pregnancy can change the gene expression profiles in the umbilical cord, and* Postn* has been shown to be one of the elevated genes that participates in the adverse metabolic effects caused by maternal obesity. In addition, Bolton et al. [21] used the signal sequence trap (SST) method in* Psammomys obesus* to identify the expression of secreted proteins in various tissues between lean, normal glucose tolerance animals (NGT group), overweight and impaired glucose tolerance animals (IGT group), and obese T2DM animals (T2DM group). There was a progressive increase in periostin expression from the NGT to the T2DM group, indicating that periostin was strongly correlated with obesity and T2DM, but the respective effects of obesity and T2DM on the expression levels of periostin remained unclear. Our study was aimed at evaluating different amounts of body fat and glucose tolerance in patients using subgroup analysis. Similarly, we observed that subjects in the OB and DM group had higher plasma periostin levels compared with the NW and NGT group, whereas the T2DM-OB subgroup had the highest levels of plasma periostin. In addition, periostin levels were positively correlated with the body fat parameters of BMI, WC, and WHR. These data suggest that obesity and impaired glucose metabolism may have a cumulative effect on the plasma levels of periostin.

Insulin resistance has been recognized as the core feature of obesity and T2DM, both of which are related to systemic inflammation [22]. Previous studies have shown that periostin is critical in regulating various inflammatory microenvironments such as airway inflammation, skin inflammation, atherosclerosis, and hepatic inflammation due to its effect on sustaining or amplifying the inflammatory responses in these pathological contexts [23]. In accord with the previous study, we found that TNF-*α* and IL-6 levels were higher in subjects with OB and T2DM. Further correlation analysis also showed a strong association between plasma periostin levels and TNF-*α* and IL-6. Considering that periostin is well involved in liver TG metabolism, while inflammation is closely related to liver TG metabolism, obesity, and diabetes [24], we may speculate that the relations between periostin, inflammation, and metabolic disorders are quite strong. As confirmed by Lu el al. [10], continuous glucose infusion via the jugular vein enhanced the expression of periostin in the liver of rats, whereas hepatic* Postn* knockdown improved the fasting glucose levels and insulin sensitivity in db/db mice. A study conducted by Amara et al. [25] suggested that the mRNA expression of periostin in HepG2 cells was increased nearly 4 times upon treating with TNF-*α*, so it is hypothesized that the high glucose state and inflammatory cytokines may contribute to the regulation of periostin biosynthesis in target tissues and that periostin may in turn decrease the insulin sensitivity. These conclusions are consistent with our study that we found a positive correlation between plasma periostin levels and variables including levels of FPG, 2 h PG, HOMA-IR, TNF-*α*, and IL-6. Moreover, further adjustment for body fat parameters slightly reduced the positive correlations among periostin, HOMA-IR, and inflammatory factors. Therefore, we speculate that obesity may involve this positive regulatory effect, and further studies are needed to clarify internal connections among periostin, HOMA-IR, and inflammation.

Several limitations should be noted in the present study. First, the sample size of our study was small. Further studies with a large sample size need to be conducted. Second, our study is cross-sectional, and thus the causality between plasma periostin levels and metabolic parameters cannot be explained.

In conclusion, the present study found that plasma periostin levels were significantly higher in T2DM and obese subjects and were strongly associated with TG metabolism, chronic inflammation, and insulin resistance. Periostin may act as a scaffold and remodeling protein in inflammatory responses, obesity, and insulin resistance, which may provide new molecular clues for the pathogenesis of T2DM and obesity.

## Figures and Tables

**Table 1:** Comparison of general clinical and laboratory parameters in the four subgroups.

Group	NGT-NW	NGT-OB	T2DM-NW	T2DM-OB
Sex (M/F)	38 (20/18)	40 (19/21)	37 (19/18)	46 (17/29)
Age	59.24 ± 8.42	58.55 ± 8.03	58.62 ± 7.92	61.13 ± 6.33
SBP (mmHg)	114.84 ± 8.10	117.95 ± 14.34	115.38 ± 8.21	114.17 ± 6.76
DBP (mmHg)	71.92 ± 6.53	70.80 ± 8.86	72.16 ± 8.10	76.48 ± 8.19^a,d,e^
Weight (kg)	52.40 ± 6.67	67.01 ± 7.40^b^	53.28 ± 7.55^d^	67.20 ± 8.16^b,f^
WC (cm)	74.68 ± 6.03	87.58 ± 6.54^b^	77.70 ± 7.74^d^	90.53 ± 7.18^b,f^
HC (cm)	89.19 ± 4.53	98.47 ± 5.13^b^	90.21 ± 4.87^c^	99.49 ± 6.15^b,f^
WHR	0.84 ± 0.05	0.89 ± 0.05^b^	0.86 ± 0.06^c^	0.91 ± 0.06^b,f^
BMI	21.54 ± 2.11	26.83 ± 1.56^b^	21.86 ± 1.90^d^	27.44 ± 2.29^b,f^
FPG (mmol/L)	5.44 ± 0.38	5.37 ± 0.40	6.62 ± 1.26^b,d^	6.95 ± 0.97^b,d^
2 h PG (mmol/L)	6.29 ± 1.24	6.48 ± 1.46	12.50 ± 2.03^b,d^	12.26 ± 1.76^b,d^
HbA1c (%)	5.79 ± 0.29	5.85 ± 0.29	6.19 ± 0.60^b,c^	6.73 ± 0.96^b,d,f^
FINS (mU/L)	4.57 ± 1.23	7.18 ± 3.29^b^	7.03 ± 4.31^a^	9.66 ± 3.82^b,c,e^
HOMA-IR	1.11 ± 0.31	1.73 ± 0.81^b^	2.13 ± 1.49^b^	3.00 ± 1.33^b,d,f^
HDL-C (mmol/L)	1.53 ± 0.42	1.28 ± 0.28^b^	1.31 ± 0.45^a^	1.13 ± 0.32^b,e^
LDL-C (mmol/L)	2.24 ± 0.71	2.48 ± 0.89	2.58 ± 0.64^a^	2.77 ± 0.68^b,e^
TC (mmol/L)	4.25 ± 1.00	4.34 ± 1.22	4.54 ± 0.86	4.91 ± 0.96^b,c^
TG (mmol/L)	1.07 ± 0.46	1.60 ± 0.83^b^	1.42 ± 0.63^b^	1.77 ± 0.61^b,e^
TNF-*α* (pg/mL)	6.42 ± 3.59	8.89 ± 3.05^b^	8.01 ± 3.48^a^	9.70 ± 3.53^b,e^
IL-6 (pg/mL)	68.08 ± 21.61	90.53 ± 31.36^b^	111.09 ± 43.23^b,d^	115.54 ± 34.13^b,d^

Data are presented as the means ± SD. Differences between multiple groups were tested by ANOVA for continuous variables. NGT, normal glucose tolerance; T2DM, type 2 diabetes mellitus; NW, normal weight; OB, obesity; SBP, systolic blood pressure; DBP, diastolic blood pressure; WC, waist circumference; HC, hip circumference; WHR, waist-hip ratio; BMI, body mass index; FPG, fasting plasma glucose; 2 h PG, 2 h postchallenge plasma glucose; HbA1c, glycated hemoglobin; FINS, fasting serum insulin; HOMA-IR, homeostasis model assessment for insulin resistance; HDL-C, high-density lipoprotein cholesterol; LDL-C, low-density lipoprotein cholesterol; TC, total cholesterol; TG, triglyceride; TNF-*α*, tumor necrosis factor *α*; IL-6, interleukin-6. ^a^ *P* < 0.05 compared with NGT-NW and ^b^ *P* < 0.01 compared with NGT-NW; ^c^ *P* < 0.05 compared with NGT-OB and ^d^ *P* < 0.01 compared with NGT-OB; ^e^ *P* < 0.05 compared with T2DM-NW and ^f^ *P* < 0.01 compared with T2DM-NW.

**Table 2:** Pearson correlation coefficient (*r*) and partial correlation of variables associated with plasma periostin.

	Plasma periostin		Plasma periostin (age- and sex-adjusted)		Plasma periostin (age-, sex-, and BMI-adjusted)	
	*r*	*P*	*r*	*P*	*r*	*P*
Age	−0.095	0.231	—	—	—	—
BMI	0.383	<0.001	0.383	<0.001	—	—
WC	0.303	<0.001	0.323	<0.001	0.073	0.363
FPG	0.310	<0.001	0.315	<0.001	0.293	<0.001
2 h PG	0.264	0.001	0.275	<0.001	0.266	0.001
HbA1c	0.204	0.01	0.199	0.012	0.120	0.132
TG	0.585	<0.001	0.594	<0.001	0.529	<0.001
LDL-C	0.089	0.264	0.120	0.132	0.085	0.288
HDL-C	−0.400	<0.001	−0.388	<0.001	−0.293	<0.001
TC	0.156	0.048	0.194	0.014	0.183	0.022
FINS	0.494	<0.001	0.483	<0.001	0.392	<0.001
HOMA-IR	0.528	<0.001	0.518	<0.001	0.434	<0.001
TNF-*α*	0.465	<0.001	0.458	<0.001	0.398	<0.001
IL-6	0.239	0.002	0.224	0.004	0.184	0.021

BMI, body mass index; WC, waist circumference; FPG, fasting plasma glucose; 2 h PG, 2 h postchallenge plasma glucose; HbA1c, glycated hemoglobin; TG, triglyceride; LDL-C, low-density lipoprotein cholesterol; HDL-C, high-density lipoprotein cholesterol; TC, total cholesterol; FINS, fasting serum insulin; HOMA-IR, homeostasis model assessment for insulin resistance; TNF-*α*, tumor necrosis factor *α*; IL-6, interleukin-6.
